# Multiscale Threats Shape the Occurrence Dynamics of a Threatened Aquatic Salamander and Reveal a Possible Extinction Debt

**DOI:** 10.1002/ece3.73225

**Published:** 2026-03-11

**Authors:** Eric W. Teitsworth, Jeffrey G. Hall, Jennifer M. Archambault, W. Jeffrey Humphries, Krishna Pacifici

**Affiliations:** ^1^ Department of Forestry and Environmental Resources North Carolina State University Raleigh North Carolina USA; ^2^ North Carolina Wildlife Resources Commission 1722 Mail Service Center Raleigh North Carolina USA; ^3^ Eastern North Carolina Ecological Services Field Office United States Fish and Wildlife Service Raleigh North Carolina USA; ^4^ Humphries Ecological LLC Carrboro North Carolina USA

**Keywords:** dynamic occupancy model, freshwater, habitat degradation, *Necturus lewisi*, urbanization

## Abstract

Freshwater ecosystems are impacted by anthropogenic stressors, resulting in roughly one‐third of freshwater fauna being threatened with extinction. The Neuse River Waterdog ( *Necturus lewisi* ) is a large aquatic salamander endemic to the Neuse and Tar‐Pamlico River basins of eastern North Carolina, USA, and it was listed as threatened under the federal Endangered Species Act in 2021. Habitat degradation has been identified as the dominant threat driving *N. lewisi* occurrence, and its effect may be delayed. The USFWS Draft Recovery Plan classified investigation into the species' occurrence dynamics (colonization/extinction) as a high priority action. We hypothesized that extinction probabilities would decrease in high quality local instream habitats, increase with high proportions of disturbed land cover in the contributing watershed, and increase in years with intense droughts. We evaluated these hypotheses within a dynamic occupancy modeling framework using five consecutive years of detection/non‐detection data collected across 176 locations. We derived estimates of annual occurrence, turnover, and equilibrium occupancy (stability) to investigate if spatial responses to threats were delayed—an extinction debt. The top‐ranked model supported the hypotheses on drivers of site‐specific extinction probabilities, including a negative effect of habitat quality, positive effect of developed land cover in the watershed, and positive effect of annual drought intensity. The derived estimates broadly indicated that annual occurrence was highest in rural subpopulations (i.e., management units), turnover was greatest in urban subpopulations, and equilibrium occupancy was lower than required to maintain stability in most subpopulations of the Neuse River basin. The estimated occurrence dynamics and their derived quantities suggested an extinction debt in urban subpopulations that may be accelerated by stochastic drought events. This study describes a novel use of the dynamic occupancy model framework within an extinction debt context and provides partnering conservation agencies with information important to guiding recovery of the Neuse River Waterdog.

## Graphical abstract

The Neuse River Waterdog ( *Necturus lewisi* ) is a threatened large aquatic salamander endemic to North Carolina, USA. Site‐specific habitat quality, the proportion of developed landcover in the watershed, and annual drought intensity operate in concert to drive the species' occurrence dynamics. Evaluation of conditional occurrence, site turnover rates, and site equilibrium (stability) highlighted regions of the species' distribution that are in or progressing towards extinction debt, informing the recovery implementation strategy of our conservation partners.

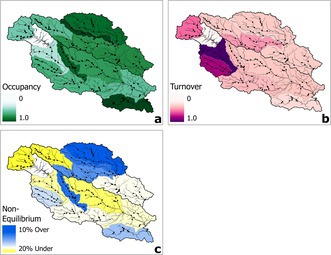

## 1 Introduction

Freshwater ecosystems disproportionately support biodiversity relative to land coverage (Dudgeon et al. 2006). These habitats are subjected to myriad human‐mediate stressors (e.g., habitat degradation, overexploitation, pollution, flow modification, and invasive species introduction), rendering roughly one‐third of freshwater fauna vulnerable to extinction, particularly those dependent upon lotic systems (Strayer and Dudgeon 2010; Collen et al. 2013). The southeastern United States is an aquatic biodiversity hotspot, harboring many freshwater species that are narrow endemics (Haag 2012; Collen et al. 2013; Richman et al. 2015). Endemic fish, crayfish, mussels, and amphibians of the southeastern US have declined in recent decades (Burkhead 2012; Adams et al. 2013; Haag and Williams 2014; Richman et al. 2015) and for many, the rate of decline remains uncertain due to a lack of data (Lynch et al. 2016; Grant et al. 2020). The biodiversity of southeastern freshwater systems is expected to continue declining in the wake of urban development, invasive species, disease, and climate change (Rothermel et al. 2008; Terando et al. 2014; Stallings et al. 2015; Grant et al. 2020).

The Neuse River Waterdog ( *Necturus lewisi* ) is a narrowly endemic, large aquatic salamander of the southeastern US that occurs exclusively in the Neuse and Tar‐Pamlico (Tar) River basins of eastern North Carolina and was historically found throughout medium to large streams of the Piedmont and Coastal Plain physiographic regions (Braswell and Ashton Jr. 1985). Females nest in coarse benthic substrates in May and June, similar to other aquatic and semi‐aquatic salamanders like *Cryptobranchus* spp. and *Eurycea* spp. (Ashton Jr. and Braswell 1979; Ashton Jr. 1985), and adults are believed to live for at least 18 years in the wild (Beane et al. 2010). *Necturus lewisi* is declining in occurrence, especially in the Neuse River basin Piedmont region, from a combination of habitat loss, land cover conversion, pollution, invasive species, dams and barriers, and climate change (USFWS 2021a). Although the species' rate of decline is unknown, a comparison of two historical surveys suggested that it occupied ~35% fewer sites in 2015 than in 1980 (NCWRC 2015). The mechanisms driving observed declines were unclear but undoubtedly influenced by the rapid expansion of the Raleigh‐Durham metropolitan area. That urban center is situated in the upper Neuse River basin and has increased ~300% in land area and human population since ca. 1980. Other putative extirpations in the upper Tar River basin appear to have been caused by extreme drought conditions in 2007–2008 (USFWS 2021a). The causes of localized occurrence loss outside of the upper Neuse and upper Tar portions of the species' range have not been clearly identified.

**FIGURE 1:**
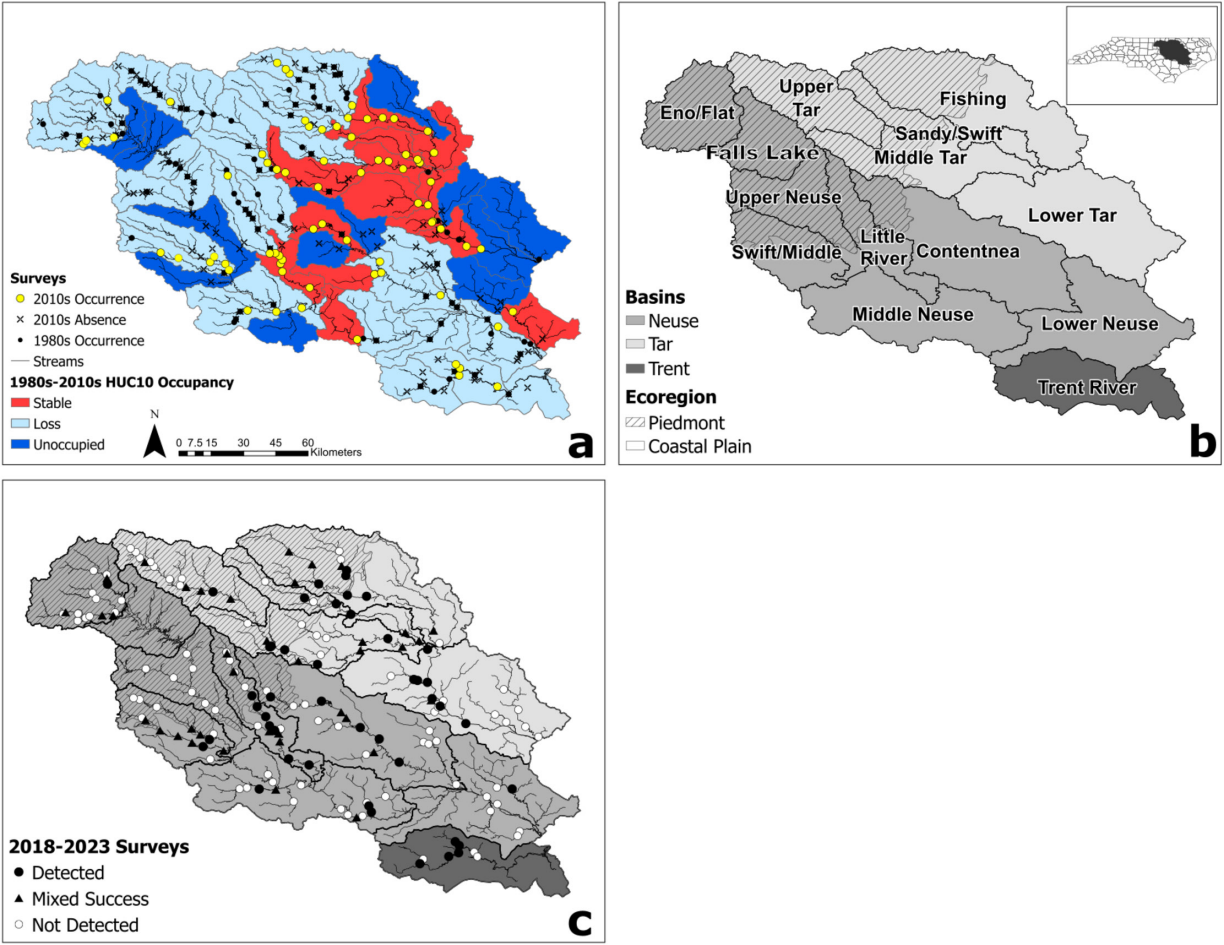
(a) Modified version of figures 3 and 4 from USFWS (2021a). Dark (filled) circles show site presence from the 1980s, while yellow circles and “X” marks show site presence and absence from the 2010s, respectively. The 1980s to 2010s change in occupancy by watershed is shown as stable (red), loss (light blue), and unoccupied (dark blue). (b) Overview of the study area where the Neuse, Tar, and Trent River subbasin are defined by different colors, the subpopulations defined by USFWS are named, and the Piedmont physiographic region is differentiated from the Coastal Plain with diagonal hatching. (c) 2018–2023 surveyed results. Dark circles represent sites with *N. lewisi* detections on every visit. Dark triangles represent sites surveyed in at least two seasons but did not have *N. lewisi* detections in every season. White (hollow) circles represent sites that never had *N. lewisi* detections. Streamlines in all panels display only permanent waterbodies of adequate size that are assumed to be suitable to *N. lewisi* (≥ 5th order using the Strahler classification).

The US Fish and Wildlife Service (USFWS) listed *Necturus lewisi* as threatened under the federal Endangered Species Act in 2021 in response to the observed declines and an increasing prevalence of assumed threats, local habitat degradation, urbanization, and climate change (USFWS 2021b; USFWS 2023; Teitsworth et al. 2024; Figure 1a). The Draft Recovery Plan (DRP) proposed that the species' recovery would require long‐term monitoring of populations and habitat, an evaluation of the magnitude of threats, and an evaluation of the species' responses to those threats across 13 recognized subpopulations (i.e., management units; USFWS 2023; Figure 1b). Prior studies with large aquatic salamanders identified local habitat quality, often substrate composition, as the significant determinant of occupancy (Durflinger Moreno et al. 2006; Quinn et al. 2013; Collins et al. 2019). Teitsworth et al. (2024) suggested that habitat degradation occurring at the local and landscape scales (i.e., sedimentation and land use change, respectively) operated in concert to drive declines in *N. lewisi* occurrence. However, none of these prior studies investigated the additive multiscale threats of local habitat degradation and urbanization, or the temporal threat of climate change on occurrence dynamics.

Often, the population‐level response to threats is delayed in long‐lived species. Tilman et al. (1994) defined the delayed loss of species occurrence following habitat destruction or alteration as “extinction debt,” where the available habitat is no longer suitable for species persistence, yet the species remains temporarily present in degraded habitats until the “debt” is paid via extirpation. Extinction debts most commonly occur in species that are large, rare, long‐lived with long generation times, have low colonization rates, are narrowly endemic habitat specialists, and whose distributions have experienced recent wide‐spread habitat destruction (Tilman et al. 1994; Hanski and Ovaskainen 2002; Kuussaari et al. 2009). Typical approaches to identifying extinction debts rely on the two‐step process of collecting demographic data and projecting the resulting age‐ or stage‐based population model forward in time (Crowder et al. 1994; Schtickzelle et al. 2005; Herben et al. 2006; Bulman et al. 2007). In this approach, the modeled metapopulations are expected to remain at equilibrium (i.e., stable state) if suitable habitat remains unchanged, but they are pushed to non‐equilibrium (e.g., growing or shrinking) as habitat suitability varies spatiotemporally (Royle and Kéry 2007).

It is often impractical to collect species' demographics data required to estimate individual turnover and therefore evaluate extinction debts for species experiencing rapid declines. However, shifting the focus from individual turnover to patch‐specific turnover (i.e., colonization/extinction) enables the evaluation of a patch‐specific extinction debt within a dynamic occupancy modeling framework, using easier to collect detection/non‐detection data (MacKenzie et al. 2003; Royle and Kéry 2007; Beissinger et al. 2022). Possible extinction debts at the patch level can be evaluated from the estimates of occurrence, turnover probabilities, and equilibrium occupancy estimates, like in the traditional metapopulation demographics context (e.g., Crowder et al. 1994), but now applied to occurrence dynamics. We could infer that an occupied patch (i.e., local population, site) is in an extinction debt if the estimated probability of annual patch turnover is high (e.g., occupied in year 1, unoccupied in year 2, occupied in year 3) and the occupancy required for patch stability is low (e.g., equilibrium occupancy > current occupancy probability).

We had two primary objectives for this study. The first objective was to identify the drivers of site‐specific occurrence dynamics of *N. lewisi* by evaluating the impact of threats at multiple scales in a dynamic occupancy modeling framework (MacKenzie et al. 2003). We hypothesized that extinction probabilities (*ɛ*) would vary by site‐level quality of local environmental features, and by landscape‐level land use/land cover (LULC) in the contributing watershed (Teitsworth et al. 2024). We also hypothesized that extinction probabilities would vary temporally with annual drought intensity (USFWS 2021a). We expected that some landscape‐level variation would be explained by stream flash flooding regimes, particularly in watersheds with higher proportions of disturbed land cover types (Konrad and Booth 2002; Walsh et al. 2005). We anticipated that initial site occupancy probabilities would increase with a combination of high local habitat quality (i.e., high scores determined during rapid habitat assessments) and high land cover quality (i.e., low proportions of developed and agricultural LULC).

The second objective was to evaluate whether subpopulations of *N. lewisi* were experiencing potential extinction debts by using estimates of occupancy, turnover probabilities, and equilibrium occupancy estimates that were derived from the parameter estimates in the top‐ranked dynamic occupancy model (USFWS 2023). We considered each subpopulation to be a discrete patch within which we could evaluate broad patterns in occurrence dynamics, and therefore extinction debt risk.

## 2 Material and Methods

### 2.1 Survey Design and Data Collection

We conducted a multi‐year survey of the historical *N. lewisi* distribution to assess the species' occurrence dynamics using a survey design that enabled estimation of initial site occupancy probabilities and colonization and extinction probabilities, while accounting for imperfect detection (MacKenzie et al. 2003). We selected survey sites using a modified stratified sample design wherein we stratified potential sites by United States Geological Survey (USGS) hydrologic watershed unit (i.e., 10‐digit code; HUC10) and prior survey data (Braswell and Ashton Jr. 1985; NCWRC 2015; Teitsworth et al. 2024). This resulted in 176 unique sites. All sites occurred in streams ≥ 5th order (i.e., Strahler classification) because streams smaller than 5th are generally unsuitable to *N. lewisi* (Braswell and Ashton Jr. 1985).

We conducted detection/non‐detection surveys November through March in five consecutive winter seasons (i.e., Years; Nov. 2018–Mar. 2019; Dec. 2019–Mar. 2020; Dec. 2020–Feb. 2021; Dec. 2021–Mar. 2022; Dec. 2022–Mar. 2023) when *N. lewisi* are most active and detection of presence is maximized (Braswell and Ashton Jr. 1985; Teitsworth et al. 2026). Not all sites were visited in every year due to logistical constraints. We visited 65 sites in year one, 75 sites in year two, 43 sites in year three, 79 sites in year four, and 72 sites in year five. We attempted to evenly distribute surveys throughout the study area each year to reduce sampling bias, however surveys in year 3 (Dec. 2020–Feb. 2021) were biased towards the Piedmont Neuse River basin sites when travel was limited due to the Covid‐19 pandemic. 68 sites were visited once (~38%), 63 sites were visited in 2 years (~36%), 34 sites were visited in 3 years (~20%), eight sites were visited in 4 years (~5%), and three sites were visited in all 5 years (~1%).

We surveyed five to eight sites concurrently each week within each year. At each site, we placed 10 Gee's Minnow Traps (Tackle Factory, Fillmore, NY) in the stream channel at 10‐m intervals and baited them with chicken liver. We set traps on Monday and checked them daily until removal on Friday for a total of 40 trap‐nights of effort per site. Bait was secured in a punctured 4‐oz plastic bottle and was not replaced unless the bottle or bait were lost. This study was approved by the North Carolina State University Institutional Animal Care and Use Committee Protocol #18‐151‐0 and #21‐326‐0.

### 2.2 Multiscale Threats

#### 2.2.1 Local Habitat Quality

We performed a single qualitative rapid habitat assessment at each site between 6 May 2021, and 2 March 2023, using standardized protocols developed by the North Carolina Department of Environmental Quality Biological Assessment Unit (NCDENR 2013). Habitat assessment protocols were weighted relative to the Piedmont or Coastal Plain physiographic regions to reflect differences in habitat features and scoring parameters between these regions, especially to account for the absence of riffle features in the Coastal Plain (NCDENR 2013). A single observer scored sites from 0 to 100 using the additive quality of eight habitat metrics, with higher scores representing higher quality habitat. Each metric was assigned a numeric score based on qualitative descriptors defined by the protocols (i.e., channel modification [0–5 Piedmont (P), 0–15 Coastal Plain (CP)], cover availability [0–20 P, CP], substrate [0–15 P, CP], pool variety [0–10 P, CP], bank erosion and vegetation [0–14 P, 0–20 CP], canopy cover [0–10, P, CP], riparian zone [0–10 P, CP], and riffle variety [0–16 P only]). Assessments were only performed in low to moderate flow conditions when the instream substrate and cover availability (e.g., rocks, logs, leafpacks, etc.) were clearly identifiable, and we assumed scores remained stable throughout the study. We recognize that the habitat may have experienced minute changes between years, but assuming stability posed minimal risk to the analysis because the rapid assessments were coarse and therefore unlikely to detect small habitat changes within the short timeframe of this study.

#### 2.2.2 Landscape Use/Cover

We sourced raster land cover data from the 2019 NLCD raster dataset and reclassified it into five broader LULC types (i.e., developed, forested, herbaceous/pasture, agricultural crops, wetland; Dewitz and US Geological Survey 2021). These land cover types were selected because developed, herbaceous/pasture, agricultural crops, and wetland LULC were estimated to have potentially important effects on site occupancy probability in a prior study (Teitsworth et al. 2024). We measured the effective proportion of each LULC type within the watershed using an inverse distance weighted approach that calculated the cumulative hydrological contribution of each type relative to its position and proportion on the landscape (Teitsworth et al. 2024). This approach allowed us to account for all LULC impacts within the watershed, while recognizing that LULC near streams had a higher relative impact than LULC far from streams. We calculated the effective proportion of each LULC type using the hydrologically active inverse flow length to stream (HAiFLS) tool in the IDW‐Plus ArcGIS custom toolset (Peterson and Pearse 2017). When dams were present in a contributing watershed, we excluded LULC above the dams from the HAiFLS calculations because we assumed dams would impound sediment and contaminant inputs from upstream sources (Bednarek 2001; Colas et al. 2011).

#### 2.2.3 Annual Drought Intensity

We determined the maximum drought experienced at each site in each year by reviewing drought intensity maps released by the US Drought Monitor (NDMC, USDA, NOAA 2023). The study area typically experiences one prolonged drought each year, if any, and multiple drought events within the same year are rare. The US Drought Monitor classifies drought intensity in six stages of increasing severity; the first four stages occurred during this study. We translated these into categorical data as follows: 0 = None, 1 = D0 (Abnormally Dry), 2 = D1 (Moderate Drought), and 3 = D2 (Severe Drought).

#### 2.2.4 Stream Discharge and Flash‐Flooding

We collected daily stream discharge data throughout the study area from USGS stream gages. Only nine of the 176 surveyed sites had a maintained USGS stream gage present within the survey segment. We interpolated average daily stream discharge m^3^/s at the other 167 non‐gaged survey sites by scaling discharge data from 31 gages distributed throughout the study area using a drainage‐area ratio.

$$Q_{u}=\frac{A_{u}}{A_{g}}*Q_{g}$$

where *A*_u_ is the drainage area of the non‐gaged site, *A*_g_ is the drainage area of the gaged site, *Q*_g_ is the discharge measured at the USGS stream gage, and *Q*_u_ is the discharge interpolated at the non‐gaged site (Hirsch 1979). We used the closest gage (i.e., ≤ 50 km) located in the same HUC10 as reference for the non‐gaged sites because the gage closest by Euclidean distance is not always the most representative of the true discharge at non‐gaged locations (Archfield and Vogel 2010; Ergen and Kentel 2016). *N. lewisi* are largely nocturnal and captures likely occurred overnight, so we expected that visit‐specific detections were influenced by discharge from the day immediately prior to the visit. When detection/non‐detection surveys were not performed at a site during a given year, we interpolated discharge values for when the surveys could have realistically been performed. These hypothetical interpolated discharge values were required for the dynamic occupancy model structure, but the model omitted these values during estimation because they were associated with “N/A” detection data. Discharge data was acquired from the USGS National Water Information System Web Interface (USGS 2023).

We used the interpolated discharge values to determine the relative flashiness of flow for each site, defined as TQ_mean_ (Konrad and Booth 2002). TQ_mean_ is the fraction of the year (i.e., number of days) that the daily mean discharge of a stream segment exceeded its annual mean discharge. Konrad (2000) found that watersheds with high proportions of impervious surfaces transported stormwater from the landscape to surface waters more efficiently, resulting in flashy stream flows and a decrease in TQ_mean_.

### 2.3 Occupancy Modeling

We formatted the detection history data for the analysis such that each survey site had zero to four visits per year in five consecutive years (maximum of 20 visits); visits occurred on four consecutive days each year and were recorded as 1 = *N. lewisi* detected or 0 = not detected. We fit a global dynamic occupancy model (i.e., one that included all additive effects; Table 1; MacKenzie et al. 2003) to the detection history data using the “unmarked” library in Program R and assessed its fit with a Goodness‐of‐Fit Test using the “AICcmodavg” library (MacKenzie and Bailey 2004; Fiske and Chandler 2011; Mazerolle 2020; R Core Team 2022).

**TABLE 1:** Covariates of detection probability, initial occupancy probability, and colonization and extinction probabilities included in the global dynamic occupancy model.

Detection probability *p*	Initial occupancy probability 𝜓	Colonization & extinction probabilities *γ*/*ϵ*
Daily Mean Discharge	Substrate Score*	Total Habitat Assessment Score*
Bait Age*	Cover Score*	Developed LULC
	Total Habitat Assessment Score*	Herbaceous/Pasture LULC
	Developed LULC	TQ_mean_
	Herbaceous/Pasture LULC	Max Annual Drought Index
	Crop LULC	
	Wetland LULC	

*Note:* Asterisks (*) denote covariates collected in the field. Covariates without asterisks were collected or derived from remotely sensed data sources.

We hypothesized that *N. lewisi* colonization and extinction probabilities would vary by site‐level habitat features (i.e., total habitat suitability) landscape‐level LULC types that are known to negatively impact site‐suitability for the species' (i.e., developed, herbaceous/pasture LULC), and temporally by annual drought intensity (USFWS 2021a; Teitsworth et al. 2024). We expected that additional variation would be introduced by stream flash flooding, particularly as it tends to increase in urban areas (Konrad and Booth 2002). To estimate variation in suitability between sites in year one, we included local‐ (i.e., substrate, cover, total habitat) and landscape‐level (i.e., developed, herbaceous/pasture, crop, and wetland LULC) covariates of initial occupancy probability that were found to be supported or potentially supported drivers of occupancy in a prior study of *N. lewisi* occurrence (Teitsworth et al. 2024). We also explored the expectation that detection probabilities would increase with increasing stream discharge and decrease as bait age increased (Chellman et al. 2017; Teitsworth et al. 2024, 2026). The effect of stream discharge on detection probability was log‐transformed in the analysis to account for the large observed range of the variable. Recapture events could theoretically obscure the accuracy of colonization/extinction/detection probability estimates if individual recapture rates were high, but recapture events accounted for < 5% of total detections in this study.

We developed models that were combinations of covariates representing the hypotheses and expectations and ranked them using AIC_c_ (Akaike's Information Criterion corrected for small sample sizes) to identify the best fit candidate model. We explored one‐way interactions between local‐ and landscape‐level covariates of initial occupancy probability. We did not explore covariate interactions in the colonization, extinction, or detection parameters of candidate models because we found that models failed to converge when those estimated parameters included interactions, likely due to the small sample size in this study. We considered models within ≤ 2.0 ΔAIC_c_ of the top‐ranked model (i.e., most parsimonious) to be equally supported and valid for drawing inference (Hurvich and Tsai 1989; Burnham and Anderson 2002).

We identified the multiscale threats driving the site‐specific occurrence dynamics of *N. lewisi* as the statistically significant covariates included in the top model (i.e., where the 95% CI did not overlap 0). We used the top‐ranked model parameter estimates to generate visit‐specific estimates of detection probability and site‐specific estimates of initial occupancy probability, annual colonization probability, and annual extinction probability. We generated annual finite‐sample estimates of occurrence at each site, which were estimates of the partially observed latent state *Z*_[*i,t*]_ that were conditioned on the observed data (i.e., 𝜓^(*fs*)^_[*i,t*]_ = 1 when presence was confirmed at site *i* in year *t*; Royle and Kéry 2007). Annual finite‐sample estimates are automatically supplied under the name of “smoothed trajectories” in a slot of dynamic occupancy models fit in “unmarked” (Weir et al. 2009; Fiske and Chandler 2011). We summarized the annual finite‐sample estimates of occurrence across all sites within each of the 13 subpopulations to investigate patterns in occurrence across the distribution of *N. lewisi* (USFWS 2023). The subpopulations specified by USFWS do not necessarily represent biologically important divisions of *N. lewisi* , but they represent the management units used by state and federal agencies, roughly follow the HUC10 watershed boundaries, and will be used in future assessments of the species' status and recovery trajectory (USFWS 2023).

### 2.4 Evaluating Extinction Debt

We evaluated possible extinction debts at each subpopulation using the summarized estimates of occurrence, turnover probability estimates, and equilibrium occupancy estimates that were derived from the top‐ranked model parameter estimates (Nichols et al. 1998; Royle and Kéry 2007). Because *N. lewisi* adults are presumably long‐lived, we expected healthy, stable subpopulations to have annual turnover probabilities near zero and annual equilibrium occupancy estimates similar to the finite‐sample estimates of occurrence (Brander et al. 2007; Royle and Kéry 2007; Beane et al. 2010). We expected that a high extinction debt risk was suggested by a turnover probability much greater than zero, and that the relative intensity of that risk was suggested by the degree of non‐equilibrium, defined here as the difference between the finite‐sample estimate of occurrence and the equilibrium occupancy estimate.

We derived summaries of the site‐specific turnover probability estimates for each subpopulation by adapting the turnover probability formula as follows:

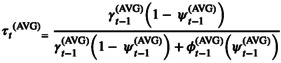

where $\gamma_{t-1}^{\left(\mathrm{AVG}\right)}$ is the average colonization probability among all surveyed sites within a given subpopulation in year *t*−1, ${\;\psi }_{t-1}^{\left(\mathrm{AVG}\right)}$ is the average occupancy probability among all surveyed sites within the subpopulation in year *t*−1, $\phi_{t-1}^{\left(\mathrm{AVG}\right)}$ is the average persistence probability (i.e., 1 − *ε*) among all surveyed sites within the subpopulation in year *t*−1, and *τ*_*t*_^(AVG)^ is the average turnover probability among all surveyed sites within the subpopulation in year *t* (Nichols et al. 1998; Royle and Kéry 2007; Kéry and Chandler 2016).

We derived summaries of the equilibrium occupancy estimates for each subpopulation by following the same logic

$${{\;\psi }_{t}^{\left(\text{AVGeq}\right)}}_{=}\;\frac{\gamma_{t}^{\left(\mathrm{AVG}\right)}}{\gamma_{t}^{\left(\mathrm{AVG}\right)}\left(\varepsilon_{t}^{\left(\mathrm{AVG}\right)}\right)}$$

where $\gamma_{t}^{\left(\mathrm{AVG}\right)}$ is the average colonization probability among sites within the given subpopulation in year *t*, $\varepsilon_{t}^{\left(\mathrm{AVG}\right)}$ is the average extinction probability among sites within the subpopulation in year *t*, and ${\;\psi }_{t}^{\left(\text{AVGeq}\right)}$ is the average equilibrium occupancy estimate among sites within the subpopulation in year *t* (Royle and Kéry 2007). We calculated the difference between the average finite‐sample estimate and average equilibrium occupancy estimate to summarize a relative measure of non‐equilibrium for each subpopulation (Brander et al. 2007). We generated a 95% confidence interval for each subpopulation‐specific estimate of average turnover probability and average equilibrium occupancy probability by parametric bootstrapping with 500 simulations.

## 3 Results

### 3.1 Survey Results

We detected *N. lewisi* (*n* = 691) at 81 sites throughout the Neuse and Tar River basins during the five‐year study (naïve *ψ ≈* 0.46; 66 historical sites and 15 new sites; Figure 1c). Nearly one half of sites with *N. lewisi* detections (*n* = 39) were visited in multiple years and had at least 1 year with no detections, possibly due to variation in daily mean discharges (see estimates in Occupancy Modeling Results). Detections occurred in free‐flowing streams with a wetted‐width of 7 to 84 m in the Piedmont and Coastal Plain physiographic regions of both basins, including the Trent River subbasin. Rapid habitat assessment scores ranged from 34 in the Neuse River in northern Johnston County to 95 in Mill Creek on the Johnston‐Wayne County line ($\bar{x}$ = 70.45 ± 12.32). Only one site with confirmed *N. lewisi* presence scored < 50 in total cumulative score. Most sites with confirmed *N. lewisi* presence scored ≥ 60 in cumulative score (*n* = 70; 86%). Higher cumulative scores indicated “better” habitat.

### 3.2 Occupancy Modeling Results

**FIGURE 2:**
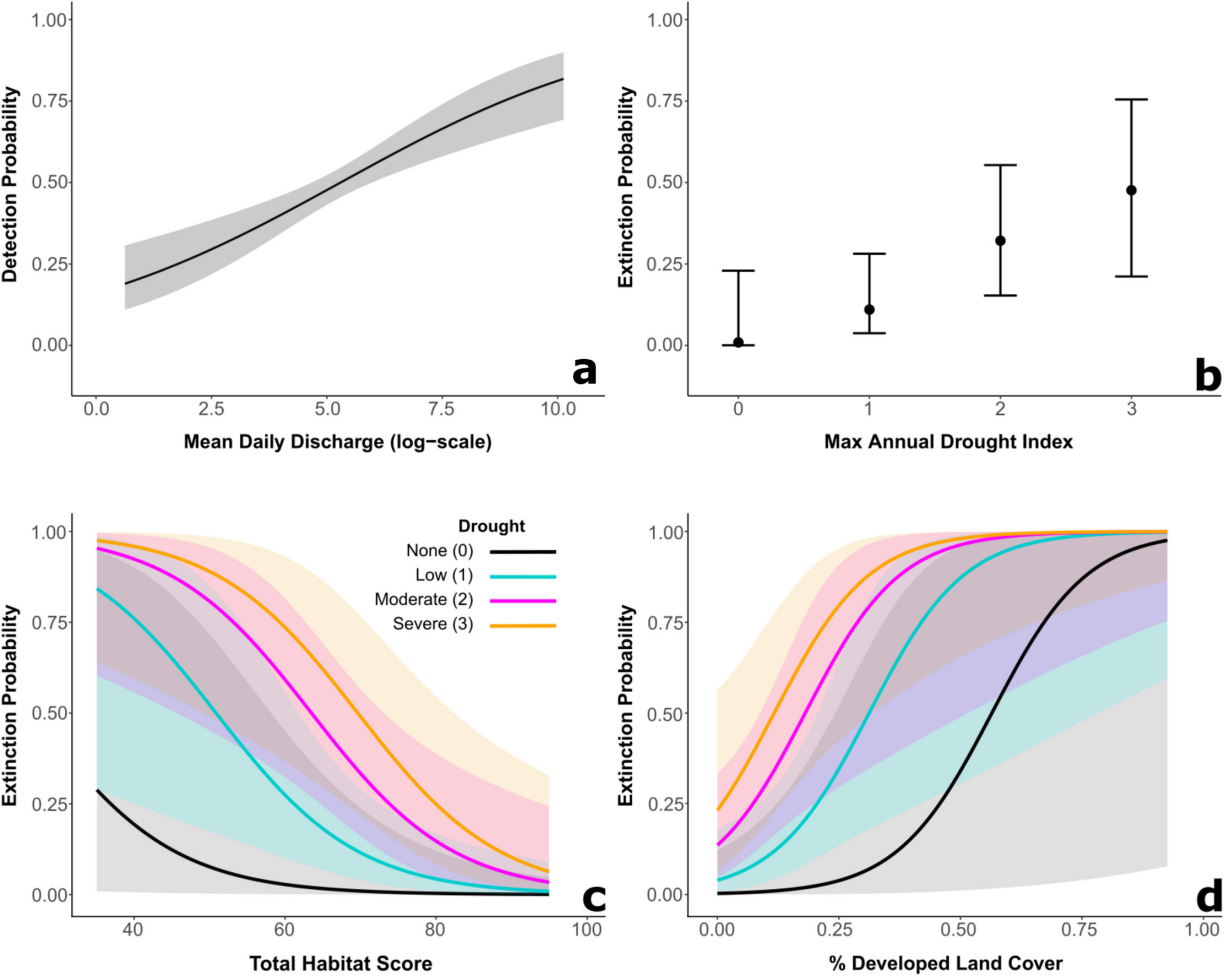
(a) Estimated effect of daily mean discharge (log‐scale) on detection probability (*p*). (b) Estimated effect of the maximum drought intensity on annual site‐specific extinction probability (*ϵ*). Solid points represent the estimated means at each drought intensity level, accounting for the effects of total habitat score and developed land cover (held at their mean effects). (c) Estimated effect of total habitat assessment score on *ϵ*, displayed at each drought intensity level and the effect of developed land cover held at its mean. (d) Estimated effect of percentage of effective developed land cover on *ϵ*, displayed at each drought intensity level and the effect of total habitat score held at its mean. The ribbons in panels a, c, and d and error bars in panel b represent the 95% confidence intervals of the estimated effects.

The Goodness‐of‐Fit Test found no evidence of overdispersion in the global model. We selected the top‐ranked candidate model for drawing inference because the second‐ranked model was 2.33 ΔAIC_c_ and only differed by one unsupported covariate (Table 2). The top‐ranked model included a positive estimated effect of total habitat assessment score as a covariate of initial site occupancy probability (𝜓; 0.183 ± 0.301 [Estimate ± SE]), but it was not well‐supported (i.e., 95% CI overlapped 0). The estimated positive effect of the log of daily mean discharge was supported as a covariate of detection probability (*p*; 0.311 ± 0.069), where higher discharge values increased the probability of detecting *N. lewisi* given true site presence (Figure 2a). The proportion of effective developed LULC was the only covariate of colonization probability (*γ*) included in the top‐ranked model. Its positive effect was not statistically supported (4.910 ± 2.983; *p* = 0.099), suggesting uncertainty in the true effect of developed land cover on site colonization probability. The top‐ranked model included the effects of maximum annual drought index (Drought 1: 2.580 ± 1.781, Drought 2: 3.930 ± 1.835, and Drought 3: 4.58 ± 1.887; Drought 0 was used as baseline; Figure 2b), total habitat assessment score (−1.290 ± 0.458; Figure 2c), and the proportion of effective developed LULC (10.23 ± 3.904; Figure 2d) as supported covariates of site extinction probability (*ε*; 95% CI did not overlap 0). There were no interactions between local and landscape effects supported by the top‐ranked model.

**TABLE 2:** Top model ranked by AIC_c_. A drought index of 0 (i.e., none) was used as the baseline for the categorical effect of drought on extinction probability.

Initial occupancy (𝜓)	Estimate	SE	CI	*p*
Total Habitat Assessment Score	0.183	0.301	(−0.41, 0.77)	0.544
Colonization (*γ*)				
Developed LULC	4.910	2.983	(−0.93, 10.76)	0.099
Extinction (*ϵ*)				
Total Habitat Assessment Score	−1.290	0.458	(−2.19, −0.39)	0.005*
Developed LULC	9.180	3.814	(2.57, 17.88)	0.016*
Drought 1	2.58	1.781	(−0.91, 6.07)	0.147
Drought 2	3.93	1.835	(0.33, 7.52)	0.032*
Drought 3	4.58	1.887	(0.88, 8.28)	0.015*
Detection (*p*)				
Daily Mean Discharge	0.311	0.069	(0.18, 0.45)	0.00001*

*Note:* Asterisks denote estimated statistical significance at *α* = 0.05.

Total habitat assessment scores had no effect on site‐specific extinction probability in years without drought. However, site‐specific extinction probabilities were substantially higher at low scoring sites in years with drought. Estimated extinction probabilities also increased at sites with > 25% developed LULC in the contributing watershed and increased considerably faster in drought years. Despite the exacerbating additive effects, the confidence intervals among the different levels of drought intensity had large overlap, suggesting uncertainty in the strength of the effect of drought alone on annual extinction probability.

Under the top‐ranked model parameter estimates, the mean detection probability was 0.476 (range across daily visits: 0.189 ± 0.050–0.818 ± 0.052), the mean initial occupancy probability was 0.466 (range across sites: 0.335 ± 0.225–0.558 ± 0.145), the mean colonization probability was constant across years and was 0.185 (range across sites: 0.109 ± 0.043–0.920 ± 0.178), and the mean estimated extinction probability was 0.285 (range across sites and years: 0.001 ± 0.001–0.999 ± 0.001). The mean finite‐sample estimates remained relatively stable across years (Year1 = 0.465, Year2 = 0.449, Year3 = 0.430, Year4 = 0.481, and Year5 = 0.454), indicating that the estimated change in annual occurrence was minimal across the species' entire distribution during this study.

**FIGURE 3:**
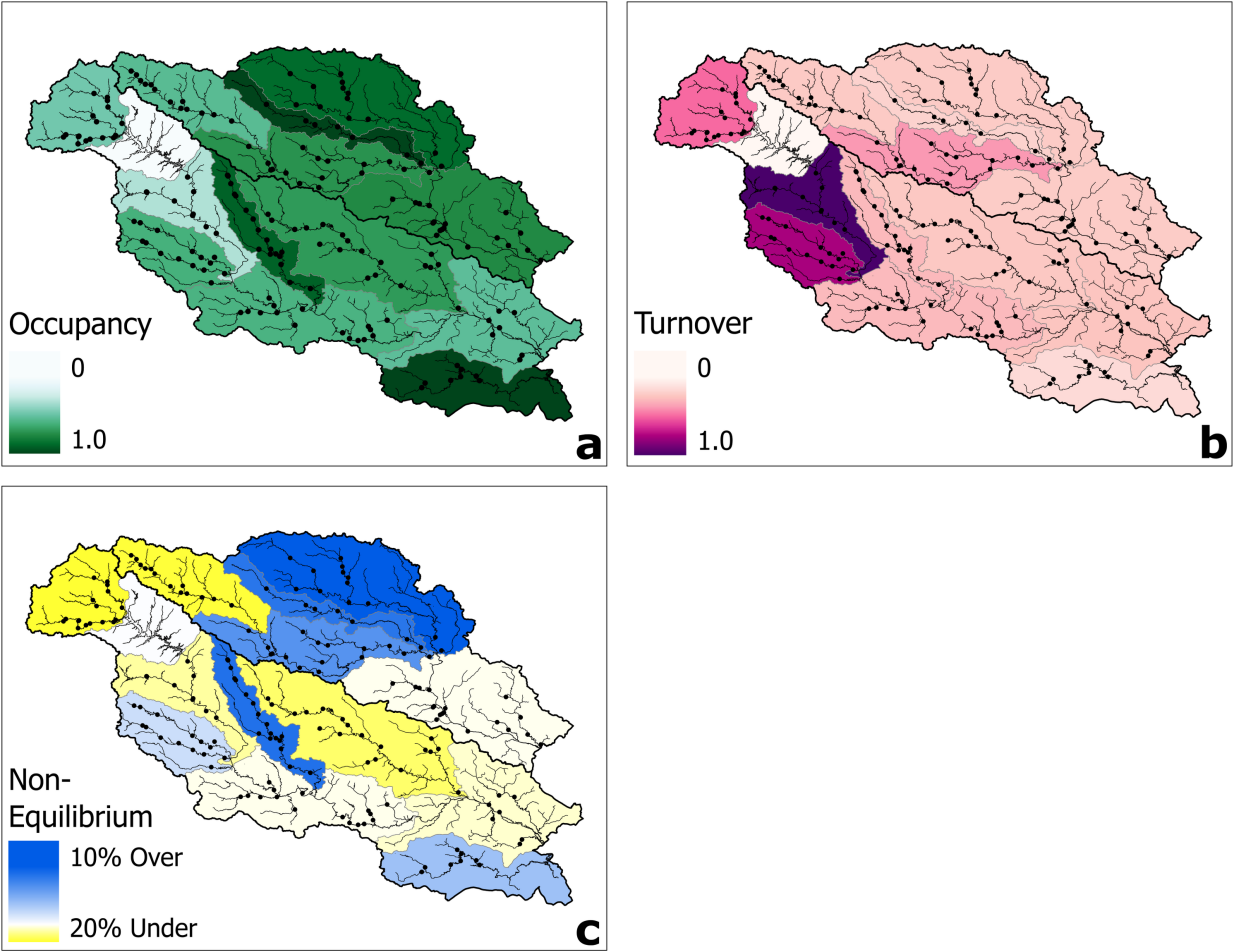
(a) Summarized mean finite‐sample estimates (i.e., conditional occupancy probability) in each USFWS subpopulation. (b) Summarized mean turnover probabilities. (c) Summarized mean non‐equilibrium value (i.e., difference between mean finite‐sample estimate and mean equilibrium occupancy estimate). The blank watershed between the Eno/Flat and Upper Neuse is a large municipal reservoir unsuitable to *N. lewisi* .

The Upper Neuse subpopulation had the lowest mean finite‐sample estimate ($\hat{\psi }$ = 0.253 [range: 0.129–0.385]). The Middle Neuse, Lower Neuse, Upper Tar, Flat/Eno, and Swift/Middle subpopulations had mean finite‐sample estimates ranging from $\hat{\psi }$ = 0.318 (0.189–0.407) in the Flat/Eno to $\hat{\psi }$ = 0.400 (0.318–0.462) in the Swift/Middle. The Contentnea Creek, Middle Tar, and Lower Tar subpopulations had mean finite‐samples estimates $\hat{\psi }$ ≈ 0.470. The Little River and Fishing Creek subpopulations had mean finite‐samples estimates $\hat{\psi }$ > 0.500. The Trent subpopulation had the highest mean finite‐sample estimate ($\hat{\psi }$ = 0.656 [0.642–0.679]), just above the Sandy/Swift subpopulation ($\hat{\psi }$ = 0.650 [0.530–0.737]; Table 3; Figure 3a).

**TABLE 3:** The subpopulation means (and range of annual means) of the finite‐sample estimates (“Occupancy”), colonization probabilities, extinction probabilities, turnover probabilities, equilibrium occupancy estimates, and the difference between the mean finite‐sample estimates and mean equilibrium occupancy estimates (“Non‐equilibrium”).

Subpopulation	Sites	Occupancy	Colonization	Extinction	Turnover	Equilibrium	Non‐equilibrium
Upper Neuse	9	0.25 (0.13–0.38)	0.30	0.55 (0.12–0.91)	0.74 (0.66–0.77)	0.28 (0.28–0.28)	−0.03
Middle Neuse	16	0.39 (0.35–0.42)	0.16	0.22 (0.08–0.44)	0.21 (0.19–0.23)	0.39 (0.38–0.42)	−0.01
Lower Neuse	7	0.36 (0.30–0.42)	0.14	0.21 (0.08–0.44)	0.19 (0.16–0.21)	0.37 (0.36–0.39)	−0.01
Flat/Eno	17	0.32 (0.19–0.41)	0.25	0.33 (0.11–0.63)	0.37 (0.31–0.40)	0.38 (0.38–0.39)	−0.06
Swift/Middle	15	0.40 (0.32–0.46)	0.34	0.44 (0.10–0.75)	0.57 (0.50–0.59)	0.36 (0.36–0.37)	0.04
Little River	18	0.61 (0.57–0.67)	0.18	0.23 (0.09–0.50)	0.22 (0.20–0.22)	0.44 (0.43–0.46)	0.17
Contentnea	19	0.46 (0.38–0.49)	0.17	0.18 (0.07–0.41)	0.18 (0.17–0.18)	0.50 (0.48–0.52)	−0.04
Trent	8	0.66 (0.64–0.68)	0.12	0.11 (0.03–0.26)	0.11 (0.10–0.12)	0.59 (0.56–0.61)	0.07
Upper Tar	13	0.36 (0.32–0.44)	0.14	0.18 (0.07–0.38)	0.17 (0.15–0.19)	0.41 (0.38–0.48)	−0.06
Middle Tar	15	0.48 (0.41–0.63)	0.18	0.27 (0.09–0.52)	0.29 (0.25–0.31)	0.36 (0.34–0.37)	0.13
Lower Tar	19	0.48 (0.45–0.52)	0.15	0.16 (0.06–0.35)	0.16 (0.16–0.17)	0.49 (0.46–0.51)	−0.01
Sandy/Swift	7	0.65 (0.53–0.74)	0.13	0.14 (0.06–0.31)	0.17 (0.15–0.18)	0.37 (0.36–0.38)	0.17
Fishing	13	0.57 (0.56–0.59)	0.12	0.20 (0.08–0.42)	0.14 (0.14–0.14)	0.48 (0.46–0.51)	0.20

*Note:* Mean colonization probabilities were constant across seasons.

### 3.3 Turnover and Equilibrium Occupancy Results

**FIGURE 4:**
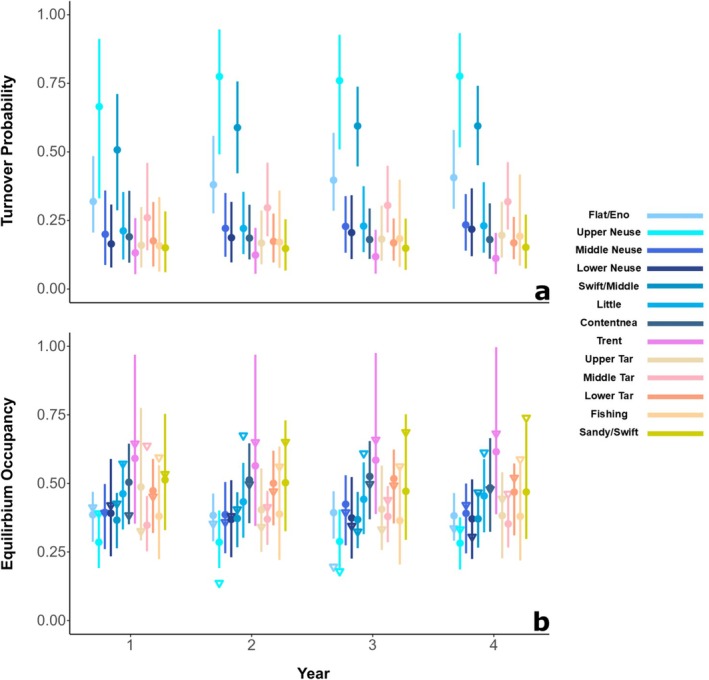
(a) Mean annual turnover probability (i.e., change in annual occupancy) in each USFWS subpopulation. (b) Mean annual equilibrium occupancy estimate (i.e., occupancy required to maintain stable state). Points represent the mean values and lines represent the 95% confidence intervals. Hollow triangles in panel b are the mean annual finite‐sample estimates. Estimates for season five are not given because occupancy dynamics and their derived quantities are not estimated for the final season in a frequentist framework (MacKenzie et al. 2003).

The turnover probability estimates indicated that there was minimal variation in annual turnover within each subpopulation (Figure 4a), but the variation was substantial among subpopulations (Figure 3b). Our expectation was that a high extinction debt risk would be suggested by a turnover probability much greater than zero, indicating a greater probability of changing occurrence status between years. The mean turnover probability was highest in the Upper Neuse subpopulation ($\hat{\tau }$ ^(AVG)^ = 0.742 [range in annual estimates: 0.662–0.775]), followed by the Swift/Middle subpopulation ($\hat{\tau }$ ^(AVG)^ = 0.567 [0.503–0.591]). Mean turnover probabilities were more moderate in the Flat/Eno ($\hat{\tau }$ ^(AVG)^ = 0.369 [0.312–0.400]), Middle Tar ($\hat{\tau }$ ^(AVG)^ = 0.288 [0.252–0.312]), Little River ($\hat{\tau }$ ^(AVG)^ = 0.215 [0.204–0.223]), and Middle Neuse subpopulations ($\hat{\tau }$ ^(AVG)^ = 0.213 [0.191–0.226]). The other seven subpopulations had relatively low mean turnover probabilities, with estimates ranging between 0.112 (Trent) and 0.185 (Lower Neuse; Table 3).

The equilibrium occupancy estimates also indicated low annual variation within subpopulations (Figure 4b) and greater variation among subpopulations (Figure 3c). We analyzed the difference between the mean finite‐sample estimate and mean equilibrium occupancy estimate for each subpopulation to assess the non‐equilibrium (i.e., instability) of each. The Flat/Eno, Upper Neuse, Upper Tar, Contentnea Creek, and Lower Neuse subpopulations had negative differences between the mean finite‐sample estimate and mean equilibrium occupancy estimate, indicating that those subpopulations had, on average, lower occupancy than what was required to maintain a stable occurrence state. The Fishing Creek, Little River, Sandy/Swift, Middle Tar, Trent, and Swift/Middle subpopulations had positive differences between the mean finite‐sample estimate and mean equilibrium occupancy estimate, indicating that those subpopulations had, on average, greater occupancy than required to maintain a stable occurrence state. The Lower Tar and Middle Neuse subpopulations had negative differences (i.e., −0.006 and −0.007, respectively), but were very close to zero, indicating that the current occurrence state was likely near equilibrium (Table 3).

## 4 Discussion

The top‐ranked model suggested that annual drought intensity (temporal), site habitat quality (local), and the proportion of effective developed LULC in the watershed (landscape) all drove site‐specific extinction probabilities for *N. lewisi* . Colonization probabilities were lower than extinction probabilities on average; however, the mean annual finite‐sample estimates remained stable during the study. The discrepancy between observed extinction events and site suitability suggested that there were possible extinction debts in the distribution of *N. lewisi* that needed additional evaluation. We summarized turnover probability estimates and equilibrium occupancy estimates within each subpopulation, expecting that summaries of these derived metrics could be used to evaluate extinction debt among subpopulations as in the metapopulation demographics context (e.g., Crowder et al. 1994; Bulman et al. 2007). By applying these approaches to an occupancy modeling framework, our results indicated that the Neuse River Piedmont subpopulations of *N. lewisi* are experiencing the highest risk of extinction debt.

Contrary to our expectations, the top model did not support specific local environmental features (e.g., substrate score, cover score) or overall habitat quality (i.e., total habitat assessment score) as significant predictors of initial occupancy probability. However, the top model supported the hypothesis that overall habitat quality was a significant covariate of site‐specific extinction probability, suggesting that poor habitat quality is a driver of extinction even if not necessarily a driver of static occurrence. Maybe *N. lewisi* (especially adults) are tolerant of poor‐quality habitat across short timespans, but population stability over longer timespans has greater sensitivity to habitat quality. Interestingly, the effect of habitat quality on extinction probability was minimal in years without drought, but drought dramatically increased site‐specific extinction probabilities at sites with poorer quality habitat. This suggests that high local habitat quality reduces the likelihood of *N. lewisi* extirpation during drought conditions.

The top‐ranked model estimated a positive effect of developed LULC on site‐specific extinction probabilities, suggesting that localized extinction events were more likely to occur at sites with high proportions of effective developed LULC in the watershed than at sites with more natural land cover types. This relationship was expected because streams in heavily developed watersheds tend to have homogenized substrates, unpredictable and flashy flow regimes, and swift extirpation of benthic‐nesting species and habitat specialists, like *N. lewisi* (Helms et al. 2005; Walsh et al. 2005; Kemp et al. 2011). It is possible that TQ_mean_ was not included in the top‐ranked model because its effect (stream flashiness) was accounted for within the broader variable of developed LULC. We also expected herbaceous/pasture LULC to influence extinction probability since prior work suggested that the proportion of herbaceous/pasture LULC in the contributing watershed may negatively affect the site‐specific occupancy of *N. lewisi* (Diana et al. 2006; Surasinghe and Baldwin 2014; Teitsworth et al. 2024), but this was not supported by the top‐ranked model. Cattle production may cause habitat degradation and reduce site suitability for *N. lewisi* in certain drainages, but chronic degradation originating from urbanization is, and will continue to be, a more pressing future concern as pastures are converted to parking lots throughout the study area (Suttles et al. 2018; Gay et al. 2023).

*Necturus* species have pronounced periods of inactivity during summer months, possibly for predator avoidance, but also possibly for thermoregulation and avoidance of high temperature and low dissolved oxygen environments (Braswell and Ashton Jr. 1985; Hutchison and Ritchart 1989; Beattie et al. 2017). Extreme droughts may limit behavioral thermoregulation and cause localized extirpation when aquatic thermal refugia are temporarily unavailable, as was suspected for the Upper Tar subpopulation of *N. lewisi* in 2007–2008 (USFWS 2021a). Drought has previously caused local extirpations of *N. lewisi* , and we suspect future drought‐related extirpations will become more frequent as drought events become more frequent and prolonged, as is predicted under some climate change models (Dai 2011). The future effect of drought is especially important to consider as drought may exaggerate the effects of poor habitat quality and developed LULC on extinction probabilities.

The turnover and equilibrium occupancy estimates both varied spatially and together provided complementary information about the extinction debt risk of each subpopulation. The turnover probability estimates suggested, on average, that the Neuse River Piedmont subpopulations exhibited the greatest amount of variation in annual site‐specific occurrence. The Upper Neuse, Flat/Eno, and Swift/Middle subpopulations exhibited the highest turnover probabilities within the species distribution. Streams within these subpopulations drain the rapidly growing Raleigh‐Durham metropolitan area. However, as evidenced by the relatively stable finite‐sample estimates during the study, average turnover did not tell the full story of extinction debt risk for this long‐lived species.

The Upper Neuse and Flat/Eno subpopulations were estimated to have slightly less occupancy than required to maintain an equilibrium state, suggesting that the elevated estimates of turnover probability were already acting on the extinction debt in these subpopulations. Conversely, the Swift/Middle subpopulation had slightly higher occupancy than required to maintain an equilibrium state, suggesting that the elevated turnover had not yet initiated an observable loss of occurrence and was further behind in the progression to extirpation. This interpretation aligns contextually with the landscape. The Swift/Middle subpopulation occurs on the periphery of the Raleigh‐Durham metropolitan area and has only recently become heavily developed, whereas the Upper Neuse and Flat/Eno subpopulations have been urbanized for decades. Investigation into the mean equilibrium occupancy estimates by subpopulation also highlighted unexpected potential extinction debts in the Upper Tar, Lower Tar, Middle Neuse, Lower Neuse, and Contentnea Creek subpopulations. Compared to the Neuse River Piedmont subpopulations, these five subpopulations were more rural and had greater habitat quality. The estimated non‐equilibrium in the Upper Tar subpopulation may be signaling the previously documented loss of occurrence caused by drought (USFWS 2023). Non‐equilibrium in the Lower Tar, Middle Neuse, Lower Neuse, and Contentnea Creek subpopulations may be tied to localized habitat degradation, such as in the headwaters of the Little Contentnea (Contentnea), Mill Creek (Middle Neuse), and Swift Creek (Lower Neuse) tributaries (NCWRC 2015; USFWS 2023; Teitsworth et al. 2024). Portions of these historically occupied subpopulations may be experiencing localized extirpations as they struggle to recolonize habitats impacted by extreme drought, agriculture, and beaver activity ( *Castor canadensis* ), contributing to an overall elevated extinction debt risk (USFWS 2023). Additional research will be needed to elucidate the causes of extinction debt risk in rural subpopulations and the potential management interventions to reverse the trend.

Occurrence dynamics are best estimated with highly standardized long‐term monitoring data collected with a view towards such analyses, but it is common for exploratory studies with rare and declining species to lack balance in survey effort (Bailey et al. 2007; Kéry and Royle 2021). The limited number of site revisits in this study may have affected the precision of the estimates of site‐specific occurrence dynamics and their supported drivers. We also acknowledge that using averages to estimate subpopulation‐specific turnover and equilibrium occupancy probabilities may have resulted in a loss of information, particularly by smoothing over natural variation between sites and years and over‐simplifying derived quantities (i.e., comparing summarized conditional data to summarized non‐conditional data; Royle and Kéry 2007). However, we favored the cursory evaluation of extinction debt across subpopulation “patches” over a finer scale evaluation to focus on the purpose of informing implementation of future recovery actions at the subpopulation scale. Future attempts to use dynamic occupancy models for extinction debt evaluation may benefit from focusing on the fine scale variation in site‐ and year‐specific derived quantities.

The chronic and intensifying effects of landscape urbanization within the Neuse and Tar River basins have caused localized environmental degradation, rendering many currently and formerly occupied sites unsuitable for *N. lewisi* . Evaluation of the occurrence dynamics and their derived quantities indicated a possible extinction debt in some subpopulations, especially those in the Neuse River Piedmont. The extinction debt in degraded subpopulations may be paid in time as the Raleigh‐Durham metropolitan areas continues to expand, and we may see range contractions where persistence is restricted to the most rural subpopulations. Localized extirpations may also be accelerated by stochastic stressors like prolonged drought events, especially in urban streams. Using a dynamic occupancy modeling approach, we now better understand the threats of drought, urbanization, local habitat degradation, and their additive effects to *N. lewisi* occurrence dynamics, and how the estimation of these threats can be leveraged to evaluate extinction debt risk in a secretive and long‐lived species. This information will help to inform the recovery implementation strategy and conservation actions of natural resource managers, aimed at reducing threats and improving populations of *N. lewisi* to support recovery. The findings of this study are being considered as stakeholders define the future goals for population persistence in a forthcoming Final Recovery Plan. We applied concepts from metapopulation theory to the approachable occupancy modeling framework to enable a cursory evaluation of a species' extinction debt risk using detection/non‐detection data, highlighting the utility of dynamic occupancy models in supporting imperiled species recovery planning and conservation.

## Author Contributions

**Eric W. Teitsworth:** conceptualization (equal), data curation (lead), formal analysis (lead), funding acquisition (equal), investigation (lead), methodology (lead), resources (equal), software (equal), supervision (lead), validation (lead), visualization (lead), writing – original draft (lead), writing – review and editing (equal). **Jeffrey G. Hall:** investigation (supporting), resources (equal), writing – review and editing (equal). **Jennifer M. Archambault:** conceptualization (equal), funding acquisition (equal), writing – review and editing (equal). **W. Jeffrey Humphries:** conceptualization (equal), funding acquisition (equal), writing – review and editing (equal). **Krishna Pacifici:** conceptualization (equal), data curation (supporting), formal analysis (supporting), funding acquisition (equal), investigation (supporting), methodology (supporting), project administration (lead), resources (equal), software (equal), supervision (supporting), validation (lead), visualization (supporting), writing – original draft (supporting), writing – review and editing (equal).

## Funding

This work was supported by the North Carolina Wildlife Resources Commission (F18AF00126) and US Fish and Wildlife Service (G21AC10267‐00).

## Conflicts of Interest

The authors declare no conflicts of interest.

## Data Availability

The data used in this study are archived and accessible in the Dryad data repository: https://doi.org/10.5061/dryad.63xsj3vgg and the GitHub data repository: https://github.com/ericteitsworth/Multiscale‐Dynamic‐Occupancy_N.‐lewisi.
